# Monitoring inflammation and airway remodeling by fluorescence molecular tomography in a chronic asthma model

**DOI:** 10.1186/s12967-015-0696-5

**Published:** 2015-10-24

**Authors:** Fabio Stellari, Angelo Sala, Francesca Ruscitti, Chiara Carnini, Prisco Mirandola, Marco Vitale, Maurizio Civelli, Gino Villetti

**Affiliations:** Chiesi Farmaceutici S.p.A, Parma, Italy; Dipartimento di Scienze Farmacologiche e Biomolecolari, Università degli Studi di Milano, Via Balzaretti 9, 20133 Milan, Italy; IBIM, Consiglio Nazionale delle Ricerche, Palermo, Italy; Dipartimento di Scienze Biomediche, Biotecnologiche e Traslazionali, Università di Parma, Parma, Italy

**Keywords:** Animal chronic asthma model, Fluorescence molecular tomography, Inflammatory cell infiltration, Airways remodeling

## Abstract

**Background:**

Asthma is a multifactorial disease for which a variety of mouse models have been developed. A major drawback of these models is represented by the transient nature of the airway pathology peaking 24–72 h after challenge and resolving in 1–2 weeks. We characterized the temporal evolution of pulmonary inflammation and tissue remodeling in a recently described mouse model of chronic asthma (8 week treatment with 3 allergens: Dust mite, Ragweed, and Aspergillus; DRA).

**Methods:**

We studied the DRA model taking advantage of fluorescence molecular tomography (FMT) imaging using near-infrared probes to non-invasively evaluate lung inflammation and airway remodeling. At 4, 6, 8 or 11 weeks, cathepsin- and metalloproteinase-dependent fluorescence was evaluated in vivo. A subgroup of animals, after 4 weeks of DRA, was treated with Budesonide (100 µg/kg intranasally) daily for 4 weeks.

**Results:**

Cathepsin-dependent fluorescence in DRA-sensitized mice resulted significantly increased at 6 and 8 weeks, and was markedly inhibited by budesonide. This fluorescent signal well correlated with ex vivo analysis such as bronchoalveolar lavage eosinophils and pulmonary inflammatory cell infiltration. Metalloproteinase-dependent fluorescence was significantly increased at 8 and 11 weeks, nicely correlated with collagen deposition, as evaluated histologically by Masson’s Trichrome staining, and airway epithelium hypertrophy, and was only partly inhibited by budesonide.

**Conclusions:**

FMT proved suitable for longitudinal studies to evaluate asthma progression, showing that cathepsin activity could be used to monitor inflammatory cell infiltration while metalloproteinase activity parallels airway remodeling, allowing the determination of steroid treatment efficacy in a chronic asthma model in mice.

**Electronic supplementary material:**

The online version of this article (doi:10.1186/s12967-015-0696-5) contains supplementary material, which is available to authorized users.

## Background

Asthma is a multifactorial and multicellular disease, and relevant animal models are needed for a better understanding of the pathophysiology of asthma and for the development of new therapeutic approaches. A variety of mouse models have been described that capture specific inflammatory mechanisms of asthma [1], but major drawbacks are represented by the transient nature of the airway pathological response, that peaks at 24–72 h after sensitization-challenge and resolve in 1–2 weeks [2, 3], and the development of tolerance after chronic exposure to single allergens [4, 5].

Recently a mouse model of chronic asthma has been described by Goplen [6], where the sensitization of Balb-C mice for 8 weeks with a combination of 3 natural allergens relevant for humans (dust mite, ragweed, and aspergillus: DRA) breaks through tolerance and establishes chronic features of asthma. Chronic DRA administration leads to inflammation and epithelial hyperplasia reaching a maximum score at the end of the sensitization protocol (week 8), with pathological changes that persist for 2–3 weeks after the last allergen exposure, even if with a lower degree of severity. Unfortunately, the conventional assessments of asthma in mice often rely on invasive measures of pulmonary function, ex vivo characterization of pulmonary cellular infiltration and observation of anatomical changes, such as airway remodeling due to inflammation, an approach that, while used extensively and highly validated, preclude the possibility of repeated, longitudinal assessment of test animals. Recently, non invasive MRI and micro-CT techniques have been applied to longitudinal monitoring of airway remodeling in murine asthma models [7, 8], and to assess lung fluid accumulation as well as the anti-inflammatory effects of S1P(1) receptor activation in allergen-induced lung inflammation [9, 10], opening the possibility to non-invasively visualize and quantitate inflammatory features of mouse asthma models in vivo.

Clinical visualization of asthma has largely been based on imaging methods such as high resolution CT, positron emission tomography (PET), single photon emission computed tomography (SPECT), and magnetic resonance imaging (MRI), that have been applied to the visualization of mechanical and physiological responses, for example diaphragmatic motion, pulmonary perfusion and ventilation as well as airway remodeling [11–14]. The techniques used in clinical imaging such as MRI, CT and PET, have been further adapted to small animal research and used in murine models of asthma [7–9, 15, 16], to serve as a preclinical, translational step between basic discovery and clinical practice, while imaging techniques developed in experimental settings may also slowly make their way into the clinical practice, especially in the context of intraoperative activities [17, 18]. Among these latter, fluorescence molecular tomography (FMT) is a relatively new tomographic imaging technique involving principles similar to X-ray CT but based on the use of target-specific molecular fluorescent reporters and volumetric reconstruction of light emitted from the probes [19].

In the present paper we report on the possibility to use near-infrared imaging agents, in combination with FMT imaging, for the non-invasive quantitative imaging of mouse lung inflammation in the DRA mouse model. The results obtained with both a cathepsin-activable and a metalloproteinase-activable fluorescent agents well correlated with ex vivo read-outs such as bronchoalveolar lavage cell analysis, pulmonary inflammatory cell infiltrations, and histological analysis of airway epithelium hyperplasia and collagen deposition, allowing differential monitoring of cellular infiltration or tissue remodeling during the inflammatory response associated with DRA.

## Methods

### Animals

Female Balb-C (7–8 week-old) mice were purchased from Charles River Laboratories Italy (Calco, Lecco, Italy). Animals were maintained under conventional housing conditions. Prior to use animals were acclimated for at least 5 days to the local vivarium conditions (room temperature: 20–24 °C; relative humidity: 40–70 %), having free access to standard mouse chow and tap water. All animal experiments described herein were approved by the intramural animal-welfare committee for animal experimentation of Chiesi Farmaceutici and comply with the European Directive 2010/63 UE, Italian D.Lgs 26/2014 and the revised “Guide for the Care and Use of Laboratory Animals” [20].

### DRA induction protocol

Mice, under light isoflurane anesthesia, were treated intranasally (i.n.) with a 30 µL volume (15 µL into each nostril) of DRA solution containing 5 µg of dust mite (extracts of *Dermatophagoides farinae,* Greer Laboratories, NC, USA), 50 µg of ragweed (extracts of *Ambrosia artemisiifolia*, Greer Lab) and 5 µg of *Aspergillus* (extracts of *Aspergillus fumigatus*, Greer Lab), twice a week for 8 weeks. The control group received 30 µL (15 µL into each nostril) of saline i.n. using the same protocol. Among the animals undergoing DRA challenge a group was treated with 100 μg/Kg budesonide i.n. 6 day/week for 4 weeks starting at the end of week 4. Eight mice were used for every group and the experiment was replicated three times.

### In vivo fluorescent imaging

DRA-challenged and control mice were injected intravenously with ProSense680 (Perkin Elmer, Hanover, CA, USA) or MMPSense680 (Perkin Elmer) at 80 nmoles/Kg on weeks 4, 6, 8 (i.e., few minutes after the last intranasal administration of DRA), and 11, and imaged 24 h after probe injection. ProSense680 is a fluorescent in vivo imaging agent that is activated by proteases such as Cathepsin B, L, S and Plasmin, and MMPSense680 is a fluorescent in vivo imaging agent that is activated by matrix metalloproteinases (MMPs) including MMP-2, -3, -9 and -13. ProSense 680 and MMPSense 680 are optically silent in its unactivated state and becomes highly fluorescent following protease-mediated cleavage.

At the 4 weeks time point, animals were randomised to budesonide and control treatments according to the intensity of the fluorescent signal resulting from ProSense680.

Mice were anaesthetized using isoflurane and depilated to minimize fur interference with fluorescent signal. Depilatory cream was applied thickly on skin over the upper torso (front, back, and sides) of each mouse, rinsed off with warm water, and reapplied until all fur had been removed.

DRA-challenged and control mice were then imaged using the FMT 2500 in vivo imaging system (VisEn Medical, Inc., Bedford, MA, USA). Briefly, the anesthetized mice were carefully positioned in the imaging cassette, which was then placed into the FMT imaging chamber. A near infrared (NIR) laser diode transilluminated (i.e., passed light through the body of the animal to be collected on the opposite side) the thorax region, with signal detection occurring via a thermoelectrically cooled charge-coupled device camera placed on the opposite side of the imaged animal. Appropriate optical filters allowed collection of both fluorescence and excitation data sets, and the multiple source-detector fluorescence projections were normalized to the paired collection of laser excitation data. The entire image acquisition sequence took approximately 4–5 min per mouse.

The collected fluorescence data were reconstructed by FMT 2500 system software version 2.2 (PerkinElmer) for the quantification of three-dimensional fluorescence signal within the lungs. Three-dimensional regions of interest were drawn in the upper torso encompassing the lung region (below the manubrium and above the liver) excluding 2 mm ventrally and 3 mm dorsally. The total amount of lung fluorescence (in picomoles) and the fluorescence volume (in cubic millimeters) were automatically calculated from the fluorescence of internal standards generated with known concentrations of appropriate NIR dyes. The same mice were submitted to both probes, and have been evaluated at 4–6–8 and 11 weeks: first the animals were injected with ProSense, and after 24–36 h MPPSense 680 was injected, after screening the residual fluorescence signal by FMT.

### Bronchoalveolar lavage fluid collection, cell counting and cytokine determination

Animals were anaesthetised with isoflurane and sacrificed by bleeding from the abdominal aorta. Eight mice were used for every time points and the experiment was replicated three times.

Bronchoalveolar lavage was performed and bronchoalveolar lavage fluids (BALFs) were collected as previously described [21]. Briefly, mouse tracheas were cannulated with a 18-gauge angiocathether. BALF was performed by gently washing the lungs with 0.6 mL sterile solution [Hank’s balanced salt solution (HBSS) × 10; (EDTA) 100 mM; 4-(2-hydroxy-ethyl)-1-piperazineethansulphonic acid (HEPES) 1 mM; distilled water] for three times in the bronchial tree and collected for subsequent analysis. After centrifugation at 400*g* for 10 min, the BAL supernatants were frozen at −80 °C. The cell pellet was re-suspended in 0.2 mL of PBS. Cell number and differential count were performed with an automatic cell counter (Dasit XT 1800J, Cornaredo, Milano, Italy). Supernatants were used for simultaneous quantitation of multiple cytokines/chemokines using a Bio-Plex™ Cytokine Assay Kit (Bio-Rad Laboratories, Segrate, Milano, Italy) according to the manufacturer instructions.

### Flow cytometric analysis of BAL cells

Twenty-four hours after the last DRA challenge, BAL was performed and cells were subsequently analyzed for surface markers. Cells were labelled in phosphate buffered saline (PBS, Euroclone) and 0.5 % bovine serum albumine (BSA, Milteny Biotech) with fluorochrome-labeled monoclonal antibodies: anti mouse CD 45 PE-Cy5 (BD Pharmigen), anti mouse F4/80 Alexa 488 (AbD Serotec), anti mouse Lys6G (BD Pharmigen), anti mouse CD11b PE-Cy7 (BD Pharmigen) and appropriate isotype controls for 30 min at RT in the dark. Cells were washed before and after the staining and resuspended in 300 µL of PBS/BSA. Samples were collected on a FACS Canto II (2 lasers, 6 colors, Becton–Dickinson) and analyzed using Diva 7 software. Mean Fluorescence Intensity (MFI) was determed on a statistically significant number of cells each sample. To positively select all leukocytes in BAL and discard debris, gating was performed on CD45 positive cells. Anti-mouse F4/80 was used to discriminate granulocyte population, including eosinophils and neutrophils, from macrophages. Lymphocytes were gated out based on forward scatter (FSC) and side scatter (SSC). Moreover anti-mouse GR1^+^ was used to negatively gate out all neutrophils. FACS analysis finally quantitated CD11b surface activation marker expression on the remaining population of monocytes/macrophages characterized as CD45^+^ F4/80^+^ GR1^−^ cells.

### Histological processing of lung and morphometric analysis

Eight mice were used for every time points and the experiment was replicated three times. Lungs were collected, gently inflated with 10 % neutral buffered formalin (about 1 mL) through a tracheal blunt needle, immersed in formalin and embedded longitudinally *en bloc*. Histological sections (3 µm) were stained with hematoxylin/eosin, alcian blue/PAS and Masson’s trichrome stains. In the haematoxylin/eosin stained sections, the following parameters were evaluated: airways epithelium hypertrophy/hyperplasia, perivascular/peribronchial/interstitial inflammation, pulmonary tissue infiltration of inflammatory cells, and vascular smooth muscle hypertrophy. Sections were semi-quantitatively scored as follows: 0, absent; 1, minimal; 2, slight; 3, moderate.

The alcian blue/PAS histochemical stain was used in order to characterize the glycoprotein component of goblet cells in the respiratory epithelium. Sections were semi-quantitatively scored, based on the amount of alcian blue/PAS positive cells observed, as follows: 0: absent/rare; 1: minimal (1–30 %); 2: slight (31–60 %); 3: moderate (>60 %).

The Masson’s trichrome histochemical stain was used to evaluate the amount of collagen deposition around the airways. Sections were semi-quantitatively scored as follows: 0, absent; 1: minimal (1–30 % of structures involved); 2: slight (31–60 % of structures involved); 3: moderate (>60 % of structures involved).

To evaluate the pulmonary inflammatory infiltrate and the airway epithelial hyperplasia in lung specimens, seven airways per mouse were taken into consideration. In order to be considered suitable for this evaluation, airways needed to be completely intact and entirely contained in a 200× microscopic field. Oblong airways were excluded from the analysis.

Pulmonary inflammatory infiltrate and airway epithelial hyperplasia, quantified in digitally acquired images analyzed with SIS software (Soft Imaging System GmbH, Münster, Germany), were measured as the area of inflammatory infiltrates and the area of epithelium (in square micrometers) divided by the airway’s basement membrane perimeter (in micrometers).

In detail, airway measurements included the perimeter of the basal membrane of the epithelium (AP), the area of the region circumscribed by the basal membrane (AA), the area of the lumen (AL) and the area covered by inflammatory infiltrates (AII). The airway epithelial hyperplasia score, corresponding to (AA − AL)/AP and the pulmonary inflammatory infiltrate score, corresponding to AII/AP, were calculated for each airway. A mouse mean score and a mean total score for each group of mice were then calculated.

### Data analysis

Data were expressed as the mean and s.e. of the mean of n animals. The inhibition values reported in different Figures represents the comparison, at a given time point, between the increase of the observed values above the dashed line (representing the average value of control animals receiving saline instead of DRA) reported in the absence and that reported in the presence of budesonide. Statistical analysis was performed using one-way ANOVA followed by Dunnett’s t test for multiple comparison (PRISM Statistical software v 4.0.3). *P* < 0.05 was considered a level of statistical significance.

## Results

**Fig. 1:**
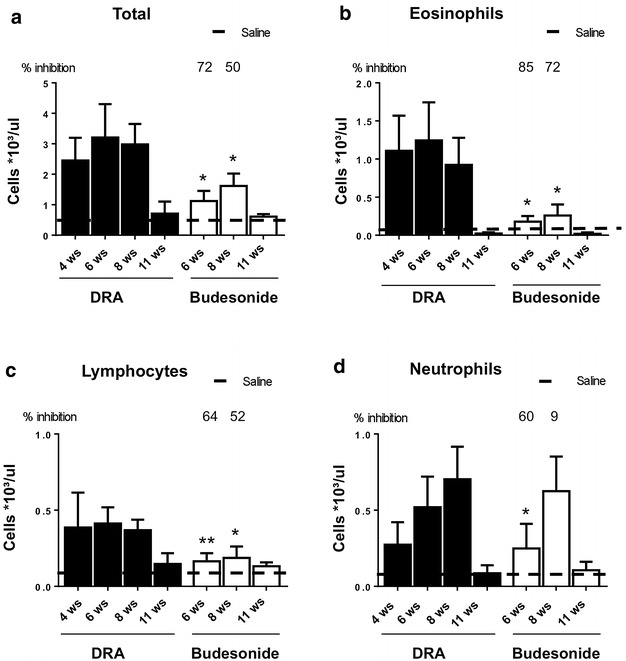
Cellular infiltration into the lung of DRA challenged mice. Total cells (**a**), eosinophils (**b**), lymphocytes (**c**) and neutrophils (**d**) infiltration into BALFs from DRA challenged mice treated with either saline or budesonide at different time points. *Values* are expressed as number of cells per μL as measured by Dasit Sysmec XT 1800. The *dashed line* represents the average value of control animals treated with saline. Eight mice were used for every time points and the experiment was replicated three times. *Values* are expressed as mean ± SEM of the three different experiments. Statistical differences were tested by one-way ANOVA followed by Dunnett’s t post hoc test for group comparisons. *P < 0.05 and **P < 0.01 vs time-matched DRA group

In agreement with the results of Goplen et al. [6], treatment with DRA for 8 weeks, twice a week, established an inflammatory pulmonary phenotype characterized by a massive inflammatory cells infiltration in the airways, as shown by the marked increase in the number of total white cells in BALFs (Fig. 1a), with eosinophils representing the prevalent population (Fig. 1b), together with lymphocytes (Fig. 1c) and neutrophils (Fig. 1d). Treatment with budesonide i.n. daily, starting at 4 weeks and continuing up to 8 weeks of DRA, markedly inhibited eosinophils and lymphocytes recruitment at 6 weeks (85 and 64 % inhibition when compared to saline-treated animals, respectively) and 8 weeks (72 and 52 % inhibition when compared to saline-treated animals, respectively) (Fig. 1b, c). The inhibition of neutrophil infiltration resulted much less pronounced and was statistically significant only at the 6 weeks time-point (Fig. 1d). Inflammatory cell infiltrate substantially resolved at 11 weeks, where the total number of cells was not statistically different from saline-treated controls.

**Fig. 2:**
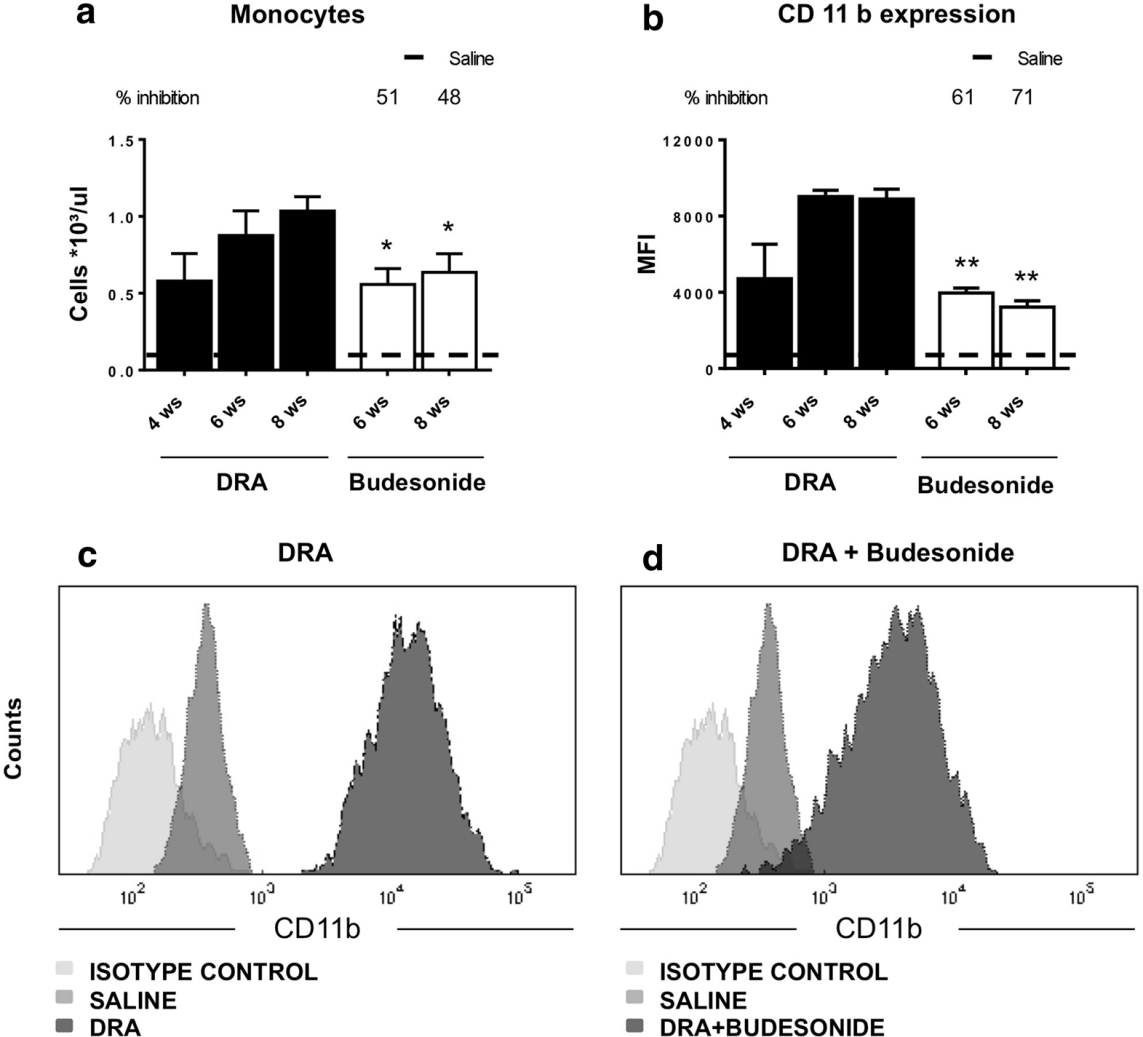
**a** Number of monocytes/macrophages in BALF from control and DRA challenged mice treated with either saline or budesonide at different time points. *Values* are expressed as number of cells per μL as measured by Dasit Sysmec XT 1800. The *dashed line* represents the average value of control animals treated with saline. **b** Expression of CD11b integrin surface marker on monocytes/macrophages present in BALFs as measured by FACS analysis in BALF from DRA challenged mice treated with either saline or budesonide at different time points. *Values* are expressed as Mean Fluorescence Intensity (MFI). The *dashed line* represents the average value of control animals treated with saline. **c** Representative picture of CD11b integrin surface marker on monocytes measured by FACS analysis; CD11b expression of mice treated with either saline (*dark grey*) or 8 weeks of DRA (*black*), with isotype control being depicted in *light grey*. **d** Representative picture of CD11b integrin surface marker on monocytes measured by FACS analysis; CD11b expression of mice treated with either saline (*dark grey*) or 8 weeks of DRA and budesonide beginning after 4 weeks of DRA (*black*), with isotype control being depicted in *light grey*. Eight mice were used for every time points and the experiment was replicated three times. *Values* are expressed as mean ± SEM of the three different experiments. Statistical differences were tested by one-way ANOVA followed by Dunnett’s t post hoc test for group comparisons. *P < 0.05 and **P < 0.01 vs time-matched DRA group

Infiltration of monocytes into the airways following DRA chronic exposure resulted in increased number of monocyte/macrophages in BALFs (Fig. 2a), with an increased expression of the integrin CD11b (Fig. 2b, c); treatment with budesonide significantly inhibited the infiltration of monocytes, reducing the number of monocytes/macrophages as well as their expression of the integrin adhesion molecule CD11b (Fig. 2a, b, d).

**Fig. 3:**
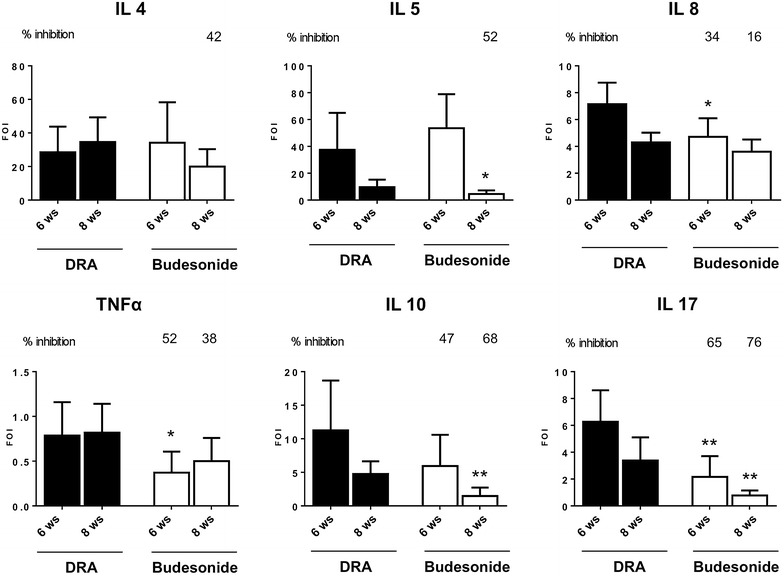
Cytokines determination in BALFs. Control and DRA challenged mice treated with either saline or budesonide were sacrificed at 6 or 8 weeks and a *panel* of 23 cytokines was analysed using a Bio-Plex™ Cytokine Assay Kit (Bio-Rad Laboratories). Eight mice were used for every time points and the experiment was replicated three times. *Values* are expressed as mean ± SEM fold-increase vs the average of the control group of the three different experiments. Statistical differences were tested by one-way ANOVA followed by Dunnett’s t post hoc test for group comparisons. P < 0.05 vs time-matched DRA group

Out of 23 cytokines evaluated in BALFs, 6 resulted elevated upon DRA administration, and budesonide statistically inhibited the increase of IL-17 both after 6 and 8 weeks of DRA. Interestingly, the inhibition of TNFα and IL-8 resulted statistically significant only after 6 weeks of DRA, while IL-5 and IL-10 concentrations were markedly affected only after 8 weeks of DRA (Fig. 3).

**Fig. 4:**
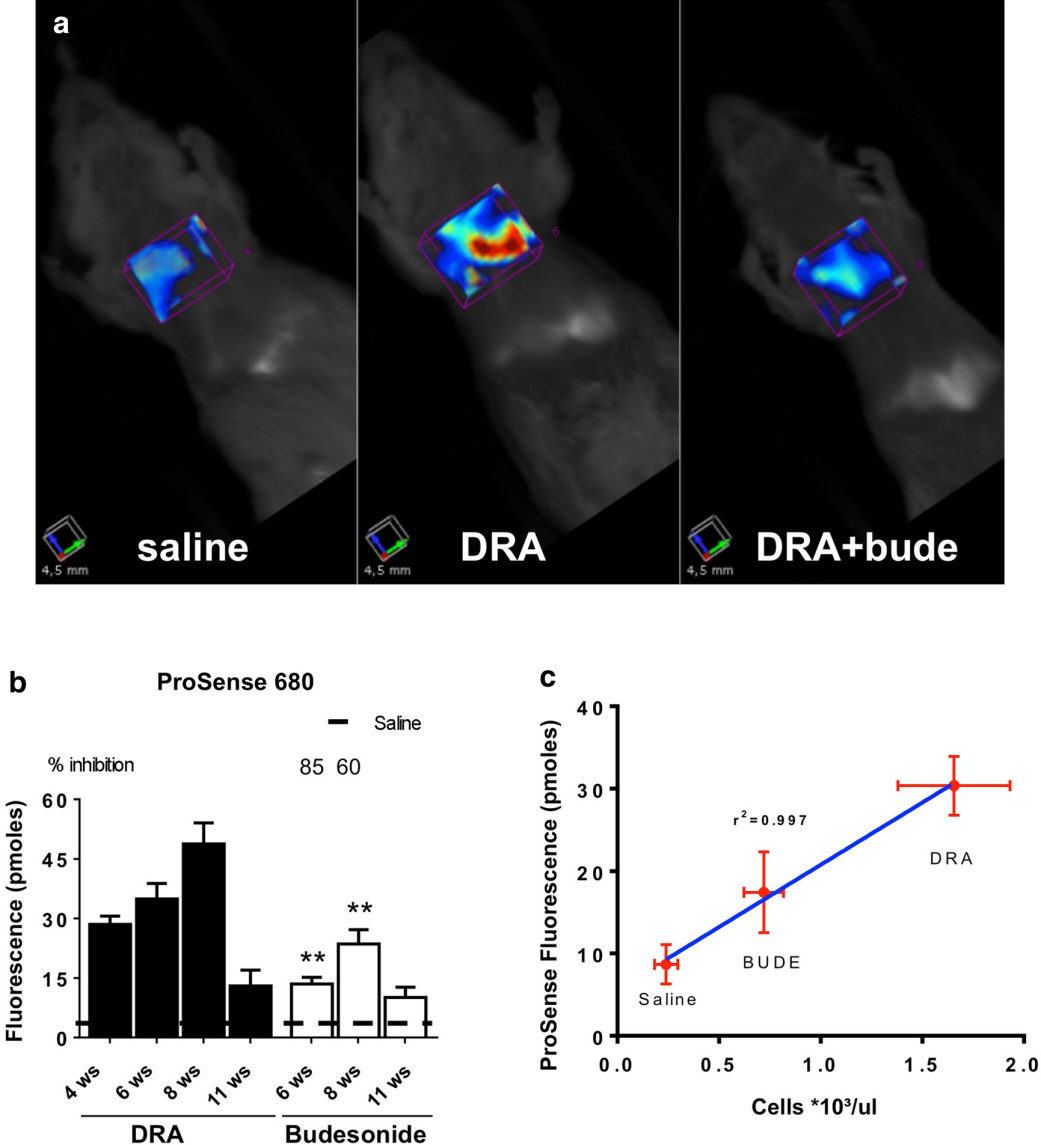
**a** In vivo imaging of representative control, DRA-challenged and DRA-challenged and budesonide-treated mice injected with Prosense680 after 8 weeks of DRA. **b** Fluorescence signal quantification measured by FMT in ProSense680-injected mice at different time points after beginning of DRA, in the absence or presence of budesonide treatment beginning after 4 weeks of DRA. *Values* are expressed as mean ± SEM of three different experiments. The *dashed line* represents the average value of control animals treated with saline. **c** Linear regression between the intensity of fluorescence resulting from ProSense680 and the total number of cell in BAL fluids from control, DRA-challenged and DRA-challenged and budesonide-treated mice injected with Prosense680 after 8 weeks of DRA. Eight mice were used for every time points and the experiment was replicated three times. *Values* are expressed as mean ± SEM of the three different experiments. Statistical differences were tested by one-way ANOVA followed by Dunnett’s t post hoc test for group comparisons. P < 0.05 and **P < 0.01

In vivo imaging of the cathepsin-activated near infrared fluorescent probe ProSense680, carried out at different times on separate groups of animals, in parallel with the groups used for BALFs fluid collection and histological analysis, showed a statistically significant increase in fluorescent signal as a result of DRA administration (Fig. 4a, b), an increase that also resulted inhibited by budesonide treatment by 85 and 60 % at 6 and 8 weeks, respectively (Fig. 4a, b). The fluorescent signal well correlated with the number of inflammatory cells in BALFs observed at 8 weeks (r^2^ 0.997; Fig. 4c), while at 11 weeks it was reduced near to basal values (Fig. 4b), in parallel with the observed decrease of inflammatory cell infiltration into the airways (Fig. 1a). *Ex vivo* imaging of excised lungs confirmed that the fluorescence observed upon ProSense and MMPSense administration was associated with the lungs (Additional file 1: Figure S1).

**Fig. 5:**
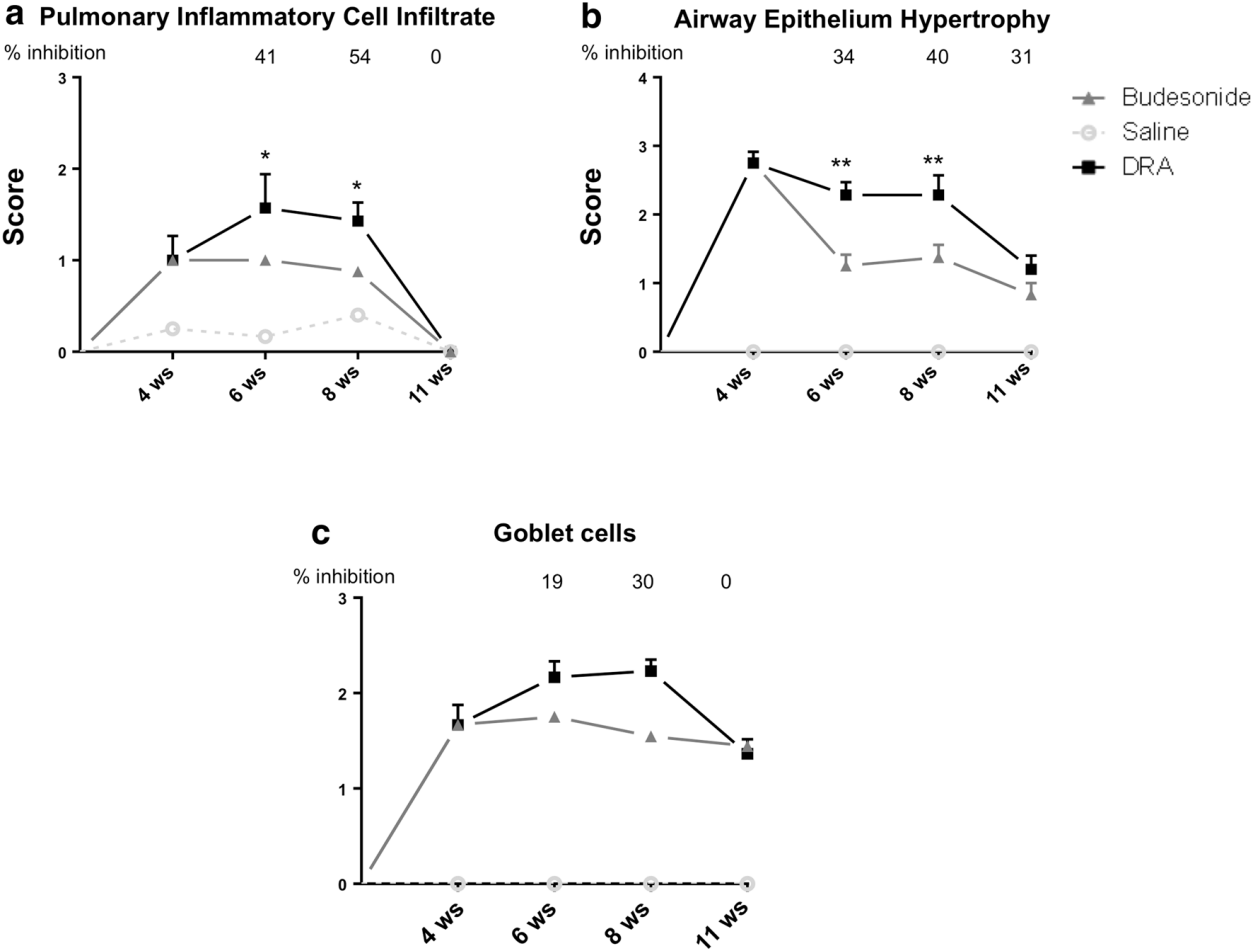
**a** Pulmonary inflammatory cell infiltrate at different time-points after the beginning of DRA in the absence or presence of budesonide treatment beginning after 4 weeks of DRA. Eight mice were used for every time points and the experiment was replicated three times. Score values, determined as described in "Methods", are expressed as mean ± SEM of the three different experiments. The *dashed line* represents the average value of control animals treated with saline. **b** Airway epithelium hypertrophy at different time-points after the beginning of DRA in the absence or presence of budesonide treatment beginning after 4 weeks of DRA. Eight mice were used for every time points and the experiment was replicated three times. Score values, determined as described in "Methods", are expressed as mean ± SEM of the three different experiments. **c** Goblet cells quantitation at different time-points after the beginning of DRA in the absence or presence of budesonide treatment beginning after 4 weeks of DRA. Eight mice were used for every time points and the experiment was replicated three times. Score values, determined as described in “Methods”, are expressed as mean ± SEM of the three different experiments. The *dashed line* represents the average value of control animals treated with saline. Statistical analysis was carried out by one-way ANOVA followed by Fischer’s exact test. P < 0.05 and **P < 0.01

Changes in the results of the histological analysis of inflammatory cell infiltrate in saline, DRA and budesonide treated animals (Fig. 5a), at different time points, well correlated with the number of inflammatory cells in BALFs as well as with the ProSense680 fluorescence signals.

**Fig. 6:**
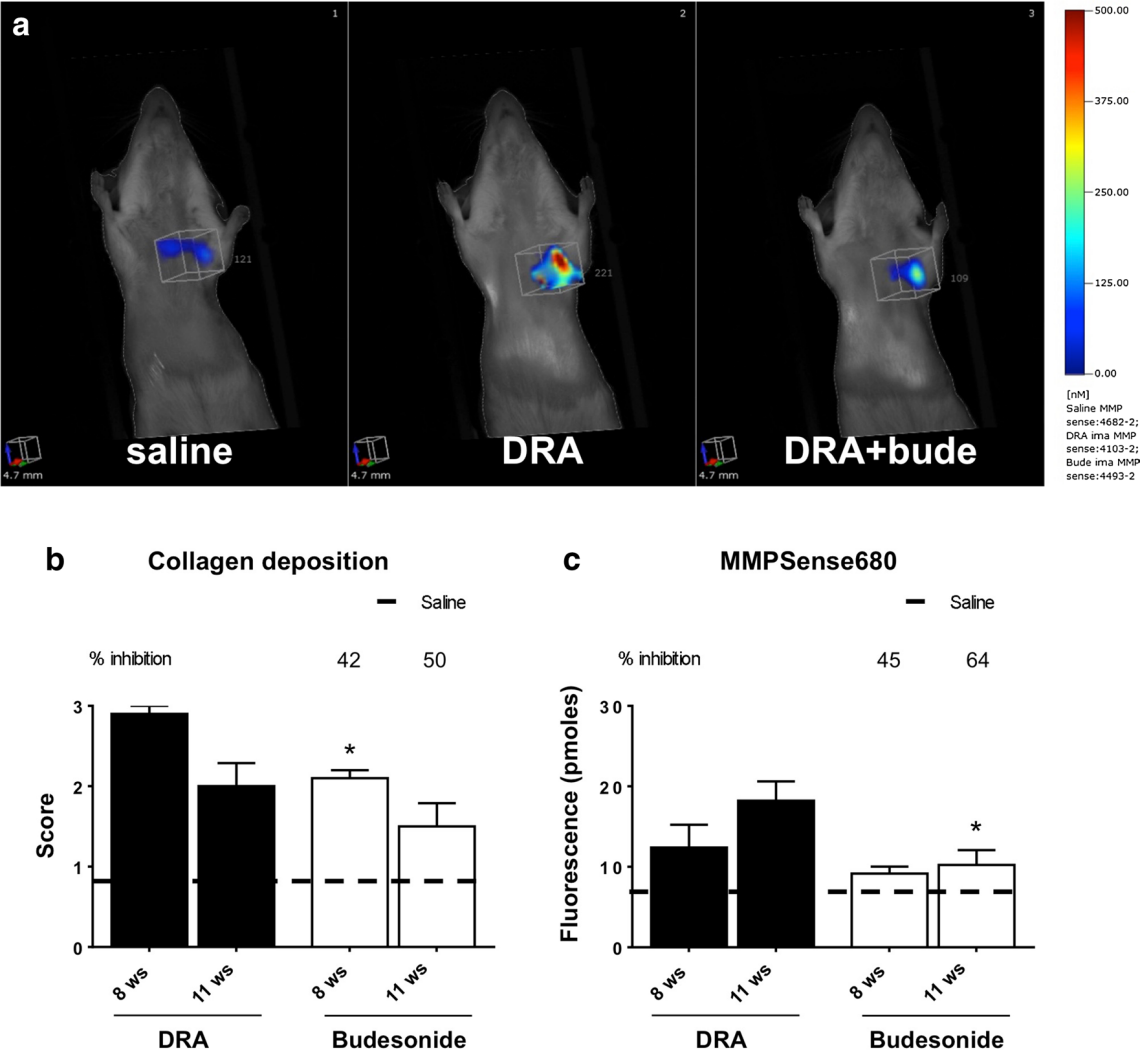
**a** In vivo imaging of representative control, DRA-challenged and DRA-challenged and budesonide-treated mice injected with MMPSense680 after 11 weeks from the beginning of DRA administration (3 weeks after ending of DRA administration). **b** Score of collagen deposition measured by Masson’s Trichrome staining at different time points after beginning of DRA, in the absence or presence of budesonide treatment beginning after 4 weeks of DRA. *Values* are expressed as mean ± SEM of three different experiments. The *dashed line* represents the average value of control animals treated with saline. **c** Fluorescence signal quantification measured by FMT in MMPSense680-injected mice at different time points after beginning of DRA, in the absence or presence of budesonide treatment beginning after 4 weeks of DRA. Eight mice were used for every time points and the experiment was replicated three times. *Values* are expressed as mean ± SEM of the three different experiments. The *dashed line* represents the average value of control animals treated with saline. Statistical differences were tested by one-way ANOVA followed by Dunnett’s t post hoc test for group comparisons. P < 0.05 and **P < 0.01

The quantitative imaging of fluorescence resulting from the metalloproteinase-activated NIRF probe MMPSense680 also showed a significant increase in DRA animals at 8 weeks, but, unlike ProSense680, it did not resolve at 11 weeks (Fig. 6a, c), when inflammatory cells were not present anymore (Fig. 1a). Although the pmoles of fluorescence signal calculated in the ROI region may produce pictures suggesting a distribution not perfectly symmetrical, this is mostly due to light scattering and we could not discriminate if a single lobe was carrying more inflammation. On the other end, histological analysis of collagen deposition at 11 weeks showed score values still significantly increased in DRA animals when compared to saline-treated animals, in agreement with the ongoing process of airways remodeling that is known to outlast inflammatory cell infiltration within the airways (Fig. 6c). A similar evolution was observed for the histological scoring of airways epithelium hypertrophy/hyperplasia, where values significantly higher than those observed in saline treated animals were still observable at 11 weeks (Fig. 5b), and similar results were observed upon quantitation of goblet cell scores (Fig. 5c). Representative histologies of Masson’s trichrome stain and Alcian/PAS of lung sections obtained after 11 weeks of DRA are presented in Additional file 2: Figure S2.

Budesonide treatment inhibited by about 50 % MMPSense680 fluorescent signal at 8 and 11 weeks (Fig. 6a, c), an effect that goes hand in hand with the observed inhibition of collagen deposition (Fig. 6b) and of the airways epithelium hypertrophy/hyperplasia (Fig. 5b).

## Discussion

In the present study we provide evidence about the possibility to monitor in vivo in mice inflammatory cells infiltration and tissue remodeling associated with repeated exposures to a combination of three natural allergens relevant for humans.

In agreement with previously published results [6] the present study confirms that multiple allergen exposure of mice breaks tolerance and induces a persisting airway pathology that is more closely mimicking the features of asthma in humans. Eight weeks of repeated allergen exposure cause a long lasting infiltration of inflammatory cells into the airways as well as pulmonary inflammation and histopathological lesions. Increased number of inflammatory cells such as eosinophils, neutrophils, monocytes/macrophages and lymphocytes were observed in BALFs and in pulmonary tissue up to 8 weeks after starting DRA administration, while collagen deposition, together with epithelial hyperplasia, were evident upon morphometric analysis at the later time points. Three weeks after stopping the exposure to allergen, cellular infiltration into the airways and the pulmonary tissue was almost completely resolved, but markers of the remodeling process such as collagen deposition, and airways epithelium hypertrophy/hyperplasia persisted, outlasting, as expected, the airways inflammatory cell infiltration [22].

Eosinophil infiltration is a hallmark of the pulmonary allergic inflammatory response, and markedly increased numbers of eosinophils were recovered in BALFs upon DRA administration. Eosinophil infiltration was very sensitive to the treatment with budesonide that strongly inhibited the increase in eosinophils both 2 and 4 weeks after the beginning of the treatment (that is after 6 and 8 weeks of DRA exposure). Interestingly, budesonide markedly decreased the concentrations of the potent maturation, differentiation and activation factor for eosinophils IL-5 [23] only at the latest time point (8 weeks of DRA) but did not affect the increased concentrations of IL-5 observed 2 weeks into the treatment with the topical corticosteroid (that is 6 week of DRA). This result supports the notion that the effects of corticosteroid treatment are not simply mediated through the prevention of IL-5 release from cells within the lung, and is in agreement with a previous observation, obtained in a different mouse model of allergic asthma, showing the effect of corticosteroids on eosinophils preceding that on the concentrations of IL-5 [24].

Repeated exposure to low-dose allergen have been reported to increase the expression of CD11b on alveolar monocytes/macrophages, consistent with an influx of monocytes [25]; DRA exposure also resulted in an increased number of monocyte/macrophages in BALFs, together with an increased expression of the adhesion molecule CD11b, as a result of a raising proportion of monocytes in the alveolar macrophage population [26, 27].

Treatment with budesonide also reduced the number of neutrophils infiltrating into the airways, but this effect was only observed at the earliest observation point (2 weeks of budesonide treatment, 6 weeks of DRA), while no inhibition was evident after 4 weeks of treatment (8 weeks of DRA). This effect well correlated with the changes in BALFs concentrations of the potent neutrophil chemotactic factor IL-8 [28], and is in agreement with previously published results [29].

Recent breakthroughs in photonic technology have allowed the advance of optical imaging beyond standard, low penetration two-dimensional fluorescence reflectance imaging (FRI) to allow for three dimensional fluorescent tomographic in vivo imaging systems. Such technology has opened the door not only to deep-tissue detection and localization but also to the new capability of absolute quantification of fluorescence at any site within the body.

This and other optical imaging techniques could also be used in animal studies to validate probes, such as peptides/antibodies that may then be used in humans by replacing the fluorescent marker with a radioisotope allowing nuclear medicine imaging [30].

Eosinophils are known to produce a variety of proteases and lysosomal cysteine proteases, including cathepsins B, L, and S [31, 32]. Such proteases may be relevant in the pathogenesis of asthma and other pulmonary diseases, through their contribution to extracellular matrix degradation [33] and the modulation of immune responses [34]. Metalloproteinase (MMP) may also play a role in pulmonary diseases such as asthma, COPD and interstitial lung diseases, where an unbalance between their activity and the activity of endogenous inhibitors (tissue inhibitors of metalloproteinases: TIMPs) is considered a key event [35–37], and MMP inhibition is an attractive therapeutic target [38]. Structural cells such as fibroblasts, bronchial and alveolar epithelial cells, and smooth muscle cells [39–41] as well as inflammatory cells such as neutrophils, alveolar macrophages, mast cells and eosinophils [42–44] are known to synthesize MMP in the lung. Taking advantage of cathepsin-sensitive (ProSense680) and metalloproteinase-sensitive (MMPSense680) near-infrared fluorescent agents associated with FMT, we monitored the activity of these proteinases within the lungs of mice at different times during the DRA administration, finding that fluorescent signal intensity well correlated with specific inflammatory responses, as assessed in parallel groups of animals. In particular, cathepsin activity provided results that quantitatively correlated with the number of inflammatory cells in BALFs, while MMP activity better correlated with remodeling processes such as collagen deposition. Although it cannot be ruled out that the correlations observed may only be circumstantial, the results obtained with post-mortem bronchoalveolar lavage (BAL) fluid analyses, cells counts, and histology at different time points all support the correlation between inflammatory cell infiltration/remodeling and the in vivo fluorescence signals observed,

Treatment with budesonide of mice undergoing DRA with already established pulmonary inflammation, decreased both the conventionally assessed inflammation parameters and cathepsin- and MMP-derived fluorescence, providing evidence that FMT allows the quantitative evaluation of the activity of corticosteroids in this recently described experimental model of asthma, both on inflammatory cells pulmonary infiltration and, at least partially, on tissue remodeling.

FMT using NIRF probes such as ProSense680 and MMSense 680 has been successfully used to assess pulmonary inflammation in LPS-treated [45, 46] and in ovalbumin-sensitized and challenged mice [46], as well as to evaluate the effect of corticosteroid in ovalbumin-sensitized and challenged mice [46, 47]. More recently, alternative NIRF probes targeting selectin adhesion molecules have also been used to monitor the recruitment of inflammatory cells in ovalbumin-sensitized and challenged mice [48, 49] and, again, the efficacy of steroids in suppressing allergic inflammatory response in the lung [49].

Nevertheless in all these studies FMT was used to monitor acute inflammatory responses, either caused by sensitization and challenge or by exposure to LPS, and/or the effect of corticosteroid treatment on such a response. In our study, the use of a recently described chronic asthma model in mice together with in vivo FMT imaging, allowed real time evaluation of activities associated with chronic allergic inflammation in the lung, enabling longitudinal non-invasive consecutive examinations.

The long lasting allergic inflammatory response observed in this model also allowed the evaluation of the therapeutic intervention with topical budesonide, rather than the prophylactic activity of corticosteroid assessed in other studies [46, 47, 49]. Indeed, randomization to treated and control groups using the FMT results of ProSense680 carried out after 4 weeks of DRA administration, minimizes variability by equally representing, in the control and budesonide-treated group, animals with different responses to DRA, therefore maximizing the possibility to assess statistically significant differences associated to treatment using a limited number of animals.

In vivo FMT imaging allowed to monitor non invasively and throughout the 11 weeks of the protocol, different component of the pulmonary inflammation associated with this novel model of chronic asthma, overcoming one of the mayor limitations for the use of asthma mouse models where conventional assessments of inflammation and asthma progression rely on invasive measures of pulmonary function and ex vivo characterization of pulmonary cellular infiltration. Although these assessments of disease status are used extensively and highly validated, they are terminal procedures that preclude the possibility of repeated, longitudinal assessment of test subjects.

## Conclusions

We studied a recently described, chronic asthma model resulting from an eight-week, twice a week, intranasal challenge with triple allergens (DRA) in female Balb/C mice. In addition to confirming that this model better reproduces the feature of human chronic asthma with inflammatory cell infiltrates associated with epithelial hyperplasia that last throughout the period of allergen administration and beyond, we also demonstrated the ability of the FMT imaging to non invasively monitor and quantify different components of the inflammatory response in the lung in a robust and consistent manner. The use of a cathepsin-sensitive agent produced results that well correlated with cellular infiltrates within the airways and the pulmonary tissue. On the other side the results obtained with a metalloproteinase-sensitive agent resulted in mirroring more closely collagen deposition and epithelial cell hypertrophy, therefore showing the potential to non-invasively monitor different component of the inflammatory response associated with DRA. The consistency of the quantitative tomography and its excellent correlation with BAL assessment and histological determinations provides a powerful tool for monitoring the disease and quantify the efficacy of therapeutic agents. FMT imaging applied to asthma research, using new and existing near infrared fluorescent imaging agents, may therefore provide a novel, non-invasive tool for understanding pulmonary inflammation and help develop new therapeutics. DRA model together with FMT will open up a new way to look at asthma progression and the pharmacological response to treatment of different classes of anti-inflammatory drugs.

## Additional files

10.1186/s12967-015-0696-5 Ex vivo imaging of fluorescence from excised lungs obtained from mice receiving saline (Panel A) or DRA (Panel B) after administration of MMPSense680 at 11 weeks and from mice receiving saline (Panel C) or DRA (Panel D) after administration of ProSense680 at 8 weeks.
10.1186/s12967-015-0696-5 Representative images of histological slides obtained at 11 weeks from the lungs of mice receiving saline (Panel A) or DRA (Panel B) stained with Alcian/PAS, and from the lungs of mice receiving saline (Panel C) or DRA (Panel D) stained with Masson’s Trichrome. Alcian/PAS and Masson’s Trichrome staining were performed as described in "Methods".

## Abbreviations

FMT: fluorescence molecular tomography
DRA: dust mite, ragweed, and aspergillus
BALFs: bronchoalveolar lavage fluids
