# Supplementary material for: Monitoring inflammation and airway remodeling by fluorescence molecular tomography in a chronic asthma model
Source: J Transl Med. 2015 Oct 24;13:336. doi: 10.1186/s12967-015-0696-5 (PMC4619338; doi:10.1186/s12967-015-0696-5)

**Figure S1.**

*Ex vivo* imaging of fluorescence from excised lungs obtained from mice receiving saline (Panel A) or DRA (Panel B) after administration of MMPSense680 at 11 weeks and from mice receiving saline (Panel C) or DRA (Panel D) after administration of ProSense680 at 8 weeks.


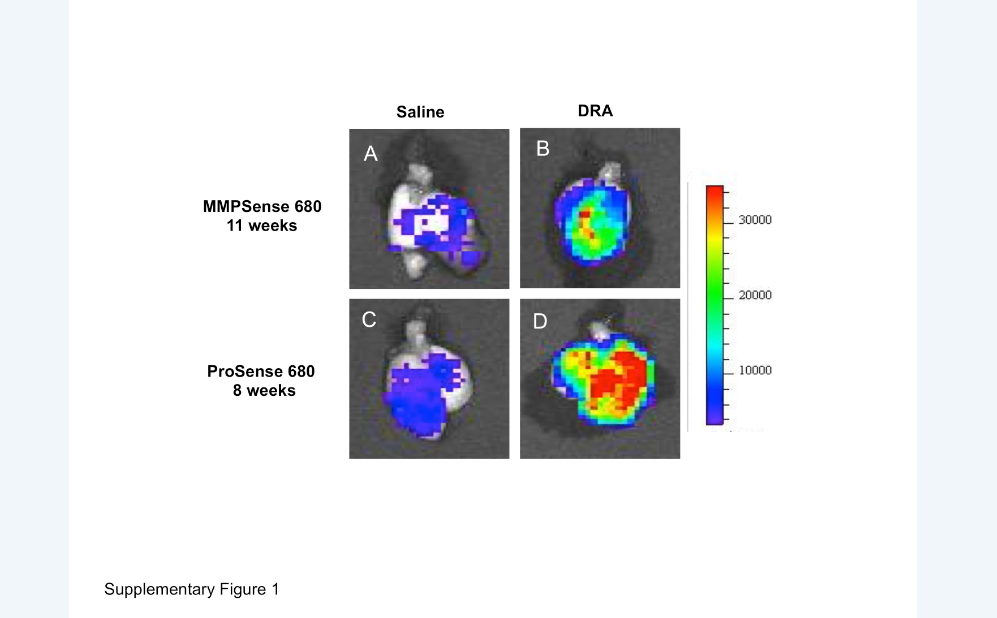

Supplement: Supplementary file 1 — 10.1186/s12967-015-0696-5 Ex vivo imaging of fluorescence from excised lungs obtained from mice receiving saline (Panel A) or DRA (Panel B) after administration of MMPSense680 at 11 weeks and from mice receiving saline (Panel C) or DRA (Panel D) after administration of ProSense680 at 8 weeks. [file 12967_2015_696_MOESM1_ESM.docx]
