# Supplementary material for: Monitoring inflammation and airway remodeling by fluorescence molecular tomography in a chronic asthma model
Source: J Transl Med. 2015 Oct 24;13:336. doi: 10.1186/s12967-015-0696-5 (PMC4619338; doi:10.1186/s12967-015-0696-5)

**Supplementary Figure 2.**

Representative images of histological slides obtained at 11 weeks from the lungs of mice receiving saline (Panel A) or DRA (Panel B) stained with Alcian/PAS, and from the lungs of mice receiving saline (Panel C) or DRA (Panel D) stained with Masson’s Trichrome. Alcian/PAS and Masson’s Trichrome staining were performed as described in Materials and Methods.


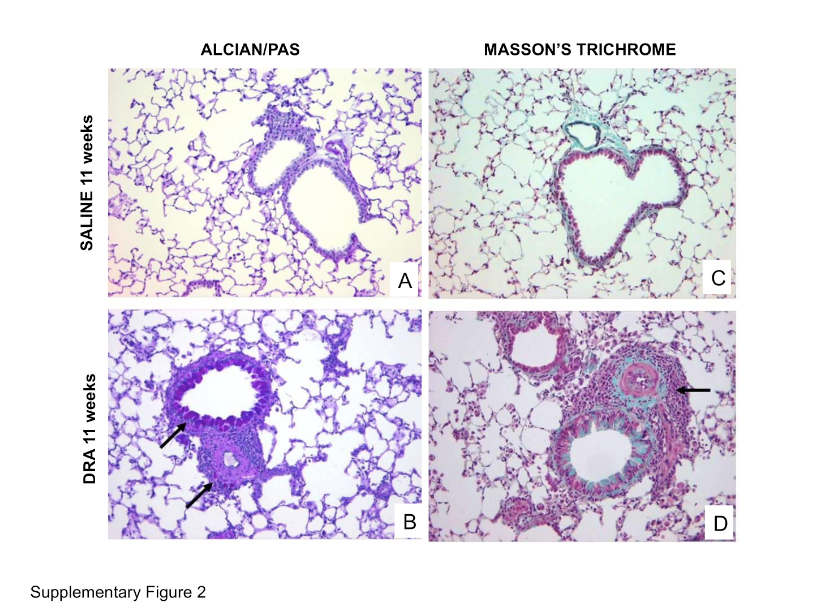

Supplement: Supplementary file 2 — 10.1186/s12967-015-0696-5 Representative images of histological slides obtained at 11 weeks from the lungs of mice receiving saline (Panel A) or DRA (Panel B) stained with Alcian/PAS, and from the lungs of mice receiving saline (Panel C) or DRA (Panel D) stained with Masson’s Trichrome. Alcian/PAS and Masson’s Trichrome staining were performed as described in "Methods". [file 12967_2015_696_MOESM2_ESM.docx]
